# First Evaluation of a Newly Constructed Underwater Pulse Oximeter for Use in Breath-Holding Activities

**DOI:** 10.3389/fphys.2021.649674

**Published:** 2021-04-26

**Authors:** Eric Mulder, Erika Schagatay, Arne Sieber

**Affiliations:** ^1^Environmental Physiology Group, Department of Health Sciences, Mid Sweden University, Östersund, Sweden; ^2^Swedish Winter Sports Research Centre, Mid Sweden University, Östersund, Sweden

**Keywords:** oxygen saturation, heart rate, apnea, validation, safety, hypoxia, hypoxic blackout, freediving

## Abstract

Studying risk factors in freediving, such as hypoxic blackout, requires development of new methods to enable remote underwater monitoring of physiological variables. We aimed to construct and evaluate a new water- and pressure proof pulse oximeter for use in freediving research. The study consisted of three parts: (I) A submersible pulse oximeter (SUB) was developed on a ruggedized platform for recording of physiological parameters in challenging environments. Two MAX30102 sensors were used to record plethysmograms, and included red and infra-red emitters, diode drivers, photodiode, photodiode amplifier, analog to digital converter, and controller. (II) We equipped 20 volunteers with two transmission pulse oximeters (TPULS) and SUB to the fingers. Arterial oxygen saturation (SpO_2_) and heart rate (HR) were recorded, while breathing room air (21% O_2_) and subsequently a hypoxic gas (10.7% O_2_) at rest in dry conditions. Bland-Altman analysis was used to evaluate bias and precision of SUB relative to SpO_2_ values from TPULS. (III) Six freedivers were monitored with one TPULS and SUB placed at the forehead, during a maximal effort immersed static apnea. For dry baseline measurements (*n* = 20), SpO_2_ bias ranged between −0.8 and −0.6%, precision between 1.0 and 1.5%; HR bias ranged between 1.1 and 1.0 bpm, precision between 1.4 and 1.9 bpm. For the hypoxic episode, SpO_2_ bias ranged between −2.5 and −3.6%, precision between 3.6 and 3.7%; HR bias ranged between 1.4 and 1.9 bpm, precision between 2.0 and 2.1 bpm. Freedivers (*n* = 6) performed an apnea of 184 ± 53 s. Desaturation- and resaturation response time of SpO_2_ was approximately 15 and 12 s shorter in SUB compared to TPULS, respectively. Lowest SpO_2_ values were 76 ± 10% for TPULS and 74 ± 13% for SUB. HR traces for both pulse oximeters showed similar patterns. For static apneas, dropout rate was larger for SUB (18%) than for TPULS (<1%). SUB produced similar SpO_2_ and HR values as TPULS, both during normoxic and hypoxic breathing (*n* = 20), and submersed static apneas (*n* = 6). SUB responds more quickly to changes in oxygen saturation when sensors were placed at the forehead. Further development of SUB is needed to limit signal loss, and its function should be tested at greater depth and lower saturation.

## Introduction

Pulse oximetry has become a well-established standard of care in clinical settings over the last few decades (Wahr and Tremper, 1995). This easy-to-use noninvasive method enables continuous monitoring of functional oxygen saturation of hemoglobin in arterial blood (SpO_2_). Currently, two different commercially available types of pulse oximeters are being used by clinicians and researchers, transmission and reflective pulse oximeters. Although these two types of devices have a different design, they both rely on the same principle for determining the oxygen saturation. Since oxyhemoglobin (O_2_Hb) and deoxyhemoglobin (HHb) have different spectral absorbance characteristics, pulse oximeters exploit this difference in light absorbance by emitting two different wavelengths of light in order to determine the proportion of hemoglobin bound to oxygen (Chan et al., 2013). Transmission pulse oximeters employ the most straight-forward technical solution by using a light emitter and detector on opposing sides of the tissue, such as the finger or the earlobe, through which light is being transmitted. In contrast, reflective pulse oximeters are built with the emitter and detector next to one another, and SpO_2_ is therefore estimated from back-scattered light (Jubran, 2015).

Conventional transmission pulse oximetry has been proven a highly accurate method to estimate arterial oxygen saturation in healthy patients under steady-state conditions (Severinghaus and Kelleher, 1992), with dropout rate for finger sensors in clinical settings of 1–2% (Barker, 2006). Initially, reflective pulse oximeters were shown to be less accurate than transmission pulse oximeters (Clayton et al., 1991), but recent technical advancements have improved the accuracy of reflective pulse oximetry considerably (Shallom et al., 2007). Under conditions of poor perfusion, however, transmission pulse oximetry at the finger may demonstrate a variable and delayed response (Severinghaus and Kelleher, 1992). This delayed response to hypoxemia is undesirable and can have disastrous outcomes, for example, in patients with pre-existing pulmonary or heart disease, or in some clinical situations when arterial oxygen saturation is expected to change quickly (Choi et al., 2010). A pulse oximeter with a short response time is therefore favorable, and a number of studies have investigated the desaturation and resaturation response times of both transmission and reflective pulse oximeters at different measurement sites during short term hypoxic events (Trivedi et al., 1997; MacLeod et al., 2005; Choi et al., 2010). Collectively, these studies confirmed that reflective pulse oximetry measured on the forehead reacted more swiftly to changes in oxygenation. Thus, during expected phases of hypoxia, reflective pulse oximetry may be the preferred monitoring method.

One non-clinical situation resulting in progressive hypoxia is breath-hold diving, also called freediving. Freediving is used by professional divers to collect food underwater (Schagatay et al., 2011), but it is also an emerging sport for recreation or competition, with pool- and deep diving disciplines (Schagatay, 2011). It is well-established from laboratory studies, using transmission pulse oximetry at the finger during voluntary breath-holding that prolonged apnea leads to a drop in SpO_2_ until approximately 30 s after apnea is terminated (Andersson and Schagatay, 1998; Andersson et al., 2002, 2004). These delays of the nadir SpO_2_ values clearly demonstrate that reflective pulse oximetry at the forehead should perhaps be used to eliminate the long response time. While this is not a major problem during steady state conditions in the lab, the real challenge for researchers lies in continuous SpO_2_ monitoring during real freediving to depth, since water- and pressure proof pulse oximeters are not commercially available. Indeed, two studies performed experimental pulse oximetry measurements at shallow depth (Stanek et al., 1993; Kuch et al., 2010), but no further advancement has been made in this development during the last decade. As this development is essential to accelerate research on freediving performance, in order to get a better understanding of human physiology in extreme conditions and to prevent freediving-related accidents, we intended to develop a new wearable datalogger to be able to measure SpO_2_ and heart rate (HR) on freedivers going to depth. As steps in this development, the aims of the current study were (I) to construct a prototype of such a system, (II) to evaluate our new prototype compared to commercial non-immersible devices during dry normoxic and hypoxic breathing conditions at rest, and (III) to conduct a pilot study on freedivers during immersed static apnea.

## Materials and Methods

The current study consisted of three parts. Part I describes the construction of a water- and pressure proof prototype reflective pulse oximeter, from here on forth referred to as “SUB pulse oximeter.” Part II aimed to evaluate SpO_2_ and HR values from two transmission pulse oximeters and the SUB pulse oximeter from participants at rest, while breathing room air, followed by inhalation of a hypoxic gas mixture. Part III aimed to compare SpO_2_ and HR patterns from one transmission pulse oximeter and the SUB pulse oximeter during static apnea with total body immersion.

### Part I: SUB Pulse Oximeter Construction

**Figure 1:**
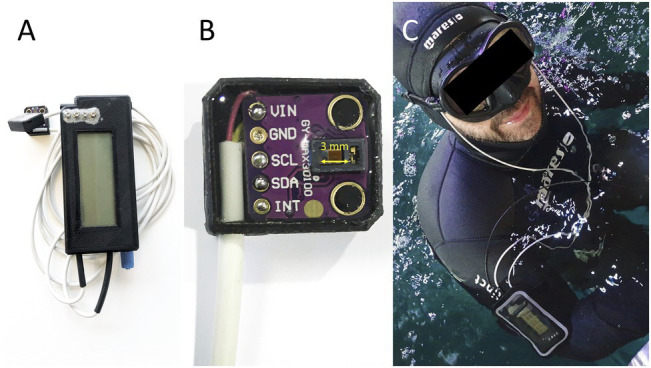
**(A)** The SUB pulse oximeter; **(B)** Close of the pulse oximetry sensor, distance between LED and photodiodes (3 mm) is marked with yellow arrow; **(C)** Freediver in the pool with the two sensor heads on the temples with the data storage unit attached to the arm.

A ruggedized and universal platform for recording of physiological, physical, or chemical parameters in challenging environments (Figure 1A) was developed within a corporation of Austrian, Croatian, Swedish, and United Kingdom teams.

#### Technical Platform

**Figure 2:**
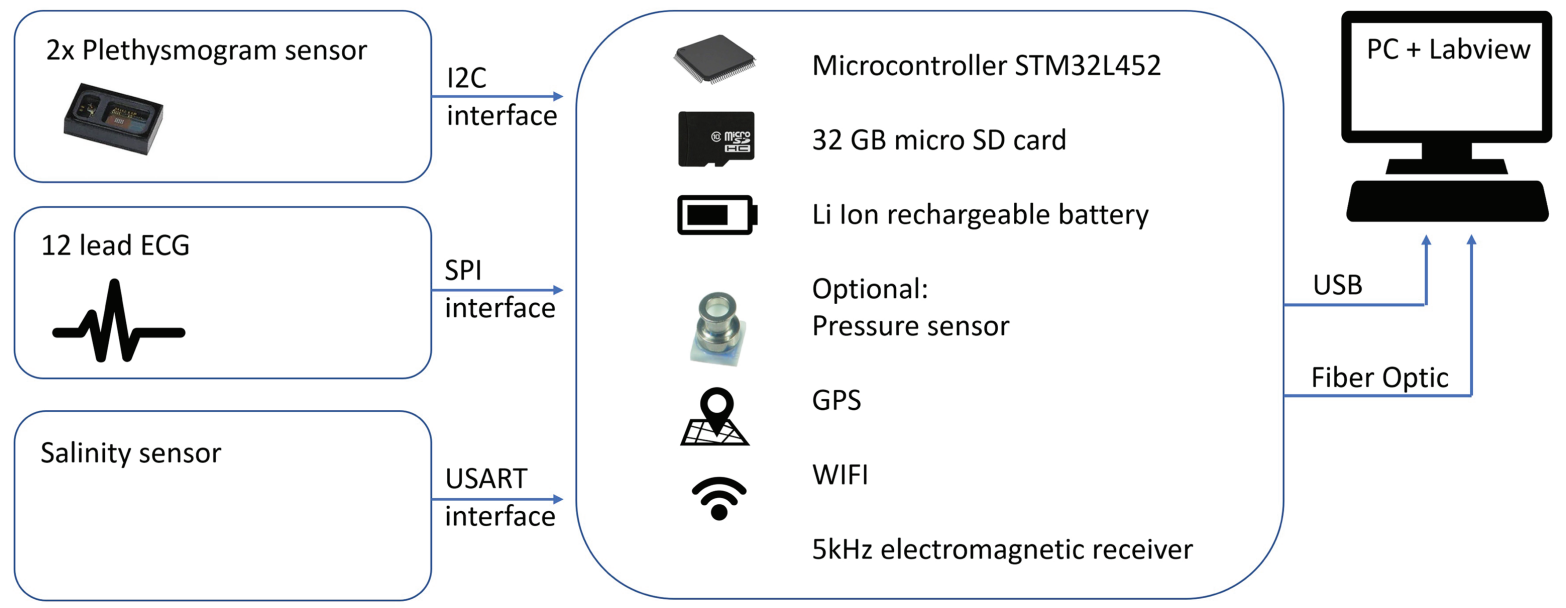
Layout of the technical platform with sensors.

The core component of the device is a ST Microelectronics STM32L452 microprocessor (STMicroelectronics International N.V. Amsterdam, The Netherlands), which is based on a 32 bit ARM Cortex M4 core. The integrated floating-point unit allows fast calculation of advanced algorithms. This processor is especially designed for low power consumption making it perfectly suitable for battery powered instrumentation. A 32 GB micro-SD card is integrated for data storage. A basic 4 × 20 characters liquid crystal display (LCD) is used to show status information of the device. The device can be connected to a USB port to charge the internal Li Ion battery and to download the recordings. The device is operated with one magnetic switch. Prototypes of the housing of the device were 3D printed. The electronics are encapsulated in silicone gel (Wacker Sil Gel 612, Wacker Chemical Corporation, MI, United States) making it dust, water, pressure, shock, and vibration proof. The microcontroller features several interfaces, which can be used to connect various sensors. A schematic overview is provided in Figure 2.

All data can be transmitted to a PC in real time with the use of either a WIFI interface based on the ESP8266 chip (Espressif Inc., Shanghai, China) or an optical fiber output. The advantage of the latter is that it can be used in water and does not require special sealing. WIFI is not suitable for underwater applications (Hollinger et al., 2011). A small screen enables real time display of the different raw variables.

#### Sensors

The device is designed as a datalogger platform. Several prototypes were developed for recording of different parameters, but for the current study focusing on SpO_2_, the prototype was equipped with two SpO_2_ probes based on the MAXIM MAX30102 chip (max 50 Hz sampling rate), temperature sensors, and an ambient pressure sensor.

#### SpO_2_ Sensors

The rapid development of the smartphone and fitness tracker industry has led to new low-cost electronic chipsets for measurements of physiological parameters in battery powered instruments. The MAX30102 (Maxim Integrated, CA, United States) sensor frontend was chosen for recording of plethysmograms. It includes red and infra-red emitters, diode drivers, photodiode, photodiode amplifier, analog to digital converter, and controller. Up to two sensor frontends can be connected to the platform, which allows synchronous recording of plethysmograms (Figures 1B,C).

#### Software Development

**Figure 3:**
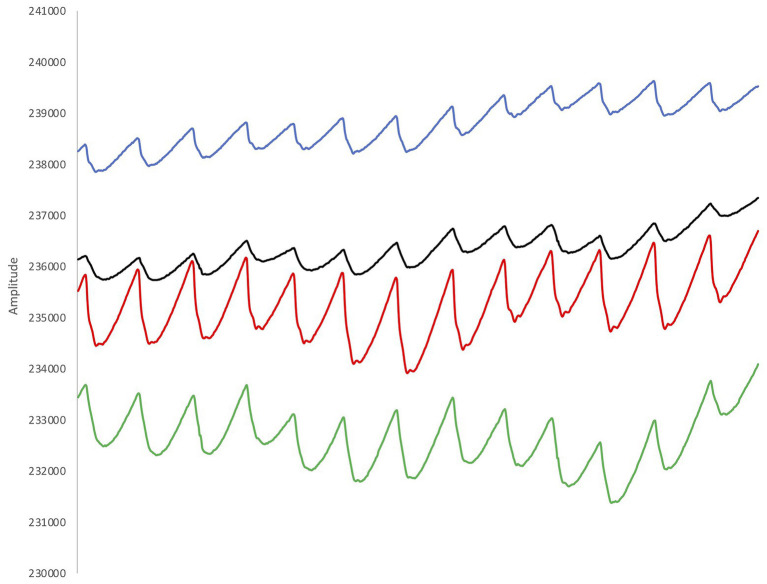
Example from the recorded plethysmograms (two signals for red light and two signals for infra-red light). The pulsatile signals show a large waveform with a sharp peak and a clear dicrotic notch, indicating good signal quality.

The firmware of the platform was developed in the programming language C and Eclipse (Eclipse Foundation, Inc., Ontario, Canada) was chosen as development environment. A graphical user interface was developed in Labview (National Instruments Corporation, Austin, United States), which can be used to show all parameters in real time and display the recordings (Figure 3). Sample algorithms for SpO_2_ calculations in low noise environments were supplied by the manufacturer (Maxim Integrated, CA, United States), however, artifacts of any kind may lead to incorrect SpO_2_ calculations. The algorithm was therefore optimized and includes an auto-correlation algorithm to filter motion artifacts. More specifically, the SpO_2_ algorithm is based on calculation of the Root Mean Square (RMS) value of alternating current (AC) and direct current (DC) of the red and infrared channel as described by the manufacturer (Application note 6,845, Maxim Integrated, CA, United States). An improved version of this algorithm was also performing a correlation between infrared and red signal to calculate a measure of signal quality. In an undisturbed signal, infrared and red signal correlate well, and in case of bad correlation, the calculated values are discarded.

### Part II and III: Evaluation of the SUB Pulse Oximeter

#### Participants

A total of 26 participants volunteered to participate in the current study, in two separate groups. Twenty healthy participants (mean ± SD age 31 ± 9 years, weight 75 ± 17 kg, height 175 ± 10 cm, 10 females) took part in part II of the study, and performed a protocol under dry circumstances. The participants in dry tests were not required to have any experience in freediving. A group of six participants (mean ± SD age 36 ± 8 years, weight 89 ± 13 kg, height 181 ± 3 cm, all males) took part in part III, and did an immersed test performed in a shallow pool. These six participants were recreational freedivers that had trained freediving for a minimum of 1 year. Safety procedures are important in all freediving related activities, so the freedivers were required to be well acquainted with safety protocols, and able to perform safety on each other. All participants gave their written informed consent to participate in the study, which was done in accordance with the Declaration of Helsinki, and approved by the Regional Committee for Medical and Health Research Ethics (Dnr 2019-05147).

#### Protocol Part II

Each volunteer was continuously monitored with two commercial transmission pulse oximeters (Lifesense LS1-9R, Nonin Medical Inc., MN, United States and Masimo Radical-7, V.7910, Masimo, Irvine, CA, United States) and the SUB pulse oximeter. To rule out any effects related to different measurement locations, we placed all pulse oximeter probes on the finger tips. Probe placement on the fingers was randomized with alternating hands among subjects. Data from all three oximeters were recorded every second. Following the placement of the probes, participants were sitting on a chair breathing room air (21% O_2_) for 5 min in order to obtain steady-state values. Then, participants were handed a rubber mouthpiece that was attached to a breathing circuit, which made sure that the participants were breathing in a hypoxic gas mixture from a Douglas bag, while breathing out through an empty tube for 3 min. The Douglas bag was filled prior to the session with a preset O_2_ and CO_2_ content (10.75% O_2_; Everest Summit I, Hypoxico Inc., NY, United States), and double-checked for its contents before and after each session using AMIS 2001 metabolic system (model C, Innovision A/S, Odense, Denmark), calibrated using a 3-L syringe and a calibration gas with 16% O_2_ and 4.5% CO_2_. The mouthpiece and an additional clip on the nose ensured that no air was inhaled beside the hypoxic gas mixture. SpO_2_ was expected to drop to no more than 75% during these 3 min, which had been established prior to the current study during pilot testing of the protocol. After 3 min, the mouth piece was removed and the participants were breathing room air and monitored until their SpO_2_ values returned to baseline.

#### Protocol Part III

**Figure 4:**
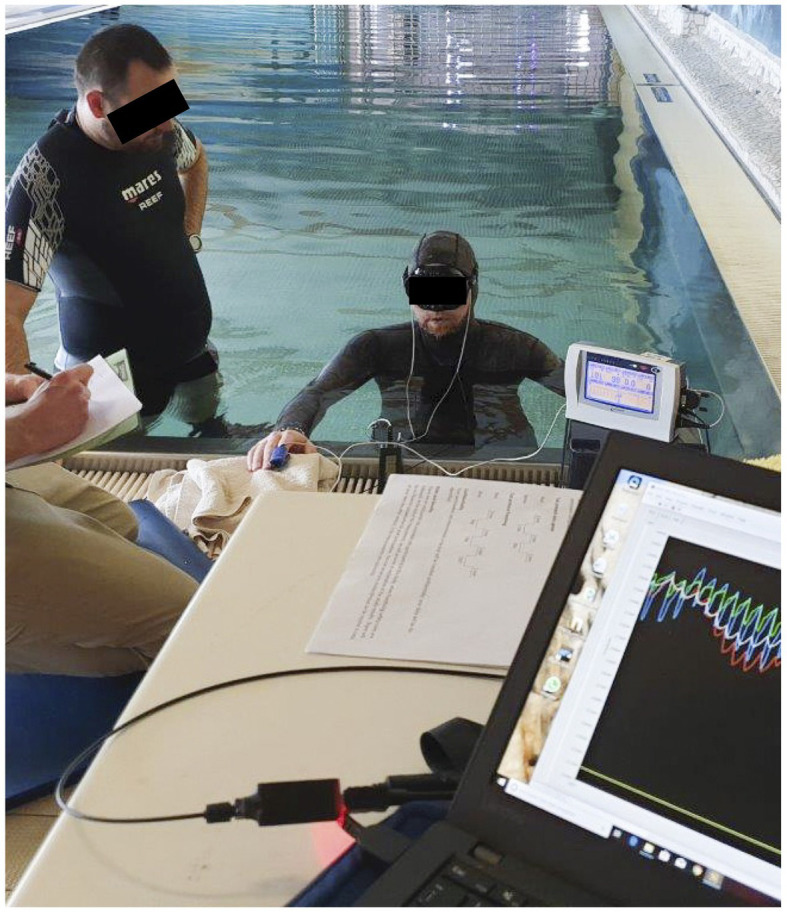
The static apnea test with the diver immersed near the surface of a pool.

The experimental procedures of the pilot tests on freedivers consisted of a static apnea test in shallow water of a pool (Figure 4). Ambient air temperature and water temperature was 32°C. The participants were asked not to eat during the last hour prior to arriving at the pool. All tests were done with the freedivers paired up in order to facilitate their usual safety procedures by breath-holding in turns, while providing safety for each other in between breath-holdings. Participants used their own mask, and neoprene wetsuit selected to provide thermal comfort in indoor pool conditions. Each volunteer was continuously monitored with a commercial transmissive finger pulse oximeter (Lifesense LS1-9R, Nonin Medical Inc., MN, United States) and the SUB pulse oximeter. Due to practical reasons, we had only one transmission finger pulse oximeter at our disposal to compare with our prototype device. Probe placement for the Lifesense pulse oximeter was the right index finger, and this hand had to remain on the (dry)side of the pool at all times. Data for the Lifesense pulse oximeter was stored on a memory unit (Trendsense, Nonin Medical Inc., MN, United States). The two sensors of the SUB pulse oximeter were placed on both temples of the diver (Figure 2B). We chose this measurement location based on prior pilot-testing, where we obtained the strongest PPG signals at the temples.

The sensors were kept in place with tape and extra fixation was provided by the pressure of the hood of the wetsuit. The pressure applied by the hood of the wetsuit was necessary to obtain more reliable SpO_2_ data, since it has previously been shown that the forehead contains a stronger venous component that the finger, and the effect of venous pulsations may deteriorate the PPG measured at the forehead (Shelley et al., 2005). Applying pressure on the sensor mechanically collapses the venous circulation and reduces the venous contribution to the signal without affecting the arterial component (Shelley et al., 2005; Agashe et al., 2006). However, to date there is no standard measure of the actual pressure applied, and too much pressure may also deteriorate the arterial component (Abey et al., 2019). We tried to solve this issue by visually inspecting the real-time PPG signals when the sensors were mounted, and when we obtained a strong signal (Figure 3), we assumed that the applied pressure was adequate. We planned to apply an extra headband in case that the pressure was not enough, but this was not necessary in any of the participants. Data was stored on the internal memory unit of the SUB pulse oximeter. The participants were not able to look at the screen of the SUB pulse oximeter, as to unable visual feedback of their physiological variables since the aim of the current experiment was not for the freedivers to push their limits with the help of visual clues, but for the investigators to observe their usual HR and SpO_2_ patterns during voluntary static apnea. Both devices were manually set to record when stable values were obtained and 2 min prior to the start of the test, and stopped recording 3 min after termination of the test. The protocol consisted of two consecutive total body immersion static apneas, i.e., the freediver was floating on the water surface in the prone position, with the face completely immersed. The first apnea functioned as a “warm-up” and had a fixed time limit of 1 min. This was followed by a 2 min breathing interval and thereafter a maximal effort apnea. Hyperventilation prior to apneas was not allowed. Participants were instructed to exhale fully and take a deep, but not maximal breath before the start of each apnea. The divers were instructed to, on command, respond with a hand signal to the safety diver to show they were doing well. The test would be interrupted if the diver failed to react immediately to avoid the development of severe hypoxia and possible risk of hypoxic syncope. Apneic duration was monitored with a stopwatch.

#### Analysis

For the SUB pulse oximeter, the mean was calculated from the results of both sensors to obtain one single value for SpO_2_ and HR for each second. The data was filtered with a 5-s running median only for part III. *Dropout rate* was presented for part II and III of the study, and refers to interruptions in continuous SpO_2_ and/or HR data due to down time or machine-probe unit nonfunction, and calculated as the percentage of time when SpO_2_ and/or HR data were not provided, as defined by Barker and Shah (1997).

For part II of the study, mean SpO_2_ and HR values from each pulse oximeter were compared from both baseline and the hypoxic challenge. For baseline, the values from 4 min to 4 min 30 s were averaged to a single SpO_2_ and HR value. For the hypoxic challenge, the values from the last 15 s were averaged to a single SpO_2_ and HR value.

In part III of the study, only the maximum apnea was used for analysis. Baseline SpO_2_ and HR for each participant were determined from the first 30 s of measurements prior to the static apnea with both pulse oximeters. For each diver, the lowest SpO_2_ value (SpO_2_ nadir) was extracted, as well as the diving HR (mean HR across the apnea minus the first 30 s). The participants performed apneas of different duration, so to enable comparison of the mean data traces we used the relative traces instead of the absolute traces. The time when SpO_2_ nadir occurred was expressed as seconds from the end of apnea, and resaturation (when values returned back to baseline) was also expressed as seconds from end of apnea.

#### Statistics

Data are presented as Mean ± SD, unless otherwise stated. A Bland-Altman analysis was used to assess the level of agreement of SpO_2_ and HR between the two transmission pulse oximeters and the SUB pulse oximeter during baseline and hypoxic episode, based on results from the 20 volunteers. Bias (the mean difference between the two methods) and precision (the SD of the bias) is presented with the 95% limits of agreement, calculated as ± 1.96 SD for the differences between the methods (Bland & Altman, 1986). Exact CIs for 95% limits of agreement based on two-sided tolerance factors were calculated (Carkeet, 2015), and presented as 97.5% outer confidence limits (OCLs) and 2.5% inner confidence limits (ICLs). For the static immersed apnea in six freedivers, mean values of HR and SpO_2_ were plotted separately, in order to compare traces from both pulse oximeters to one another.

## Results

All pulse oximeters functioned properly in part II and concomitantly the signal dropout rate was <1%. In part III, the signal dropout rate for the SUB pulse oximeter was 18% for the raw data and 1% after applying the averaging function for all trials, while it was <1% for the transmission pulse oximeter.

### Part II: Evaluation of SpO_2_ and HR During Baseline and Hypoxic Episode

**Figure 5:**
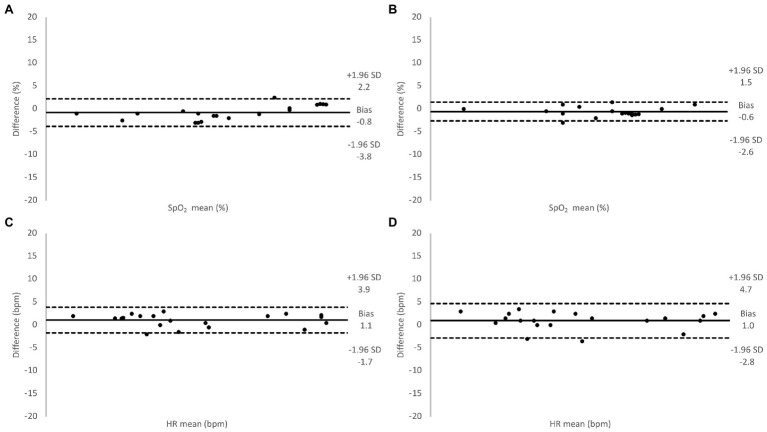
Relative difference between oxygen saturation (SpO_2_) values measured using the Masimo pulse oximeter and the SUB pulse oximeter **(A)**, the Lifesense pulse oximeter and the SUB pulse oximeter **(B)**, and for HR values measured using the Masimo pulse oximeter and the SUB pulse oximeter **(C)**, the Lifesense pulse oximeter and the SUB pulse oximeter **(D)**, under normoxic conditions.

**Figure 6:**
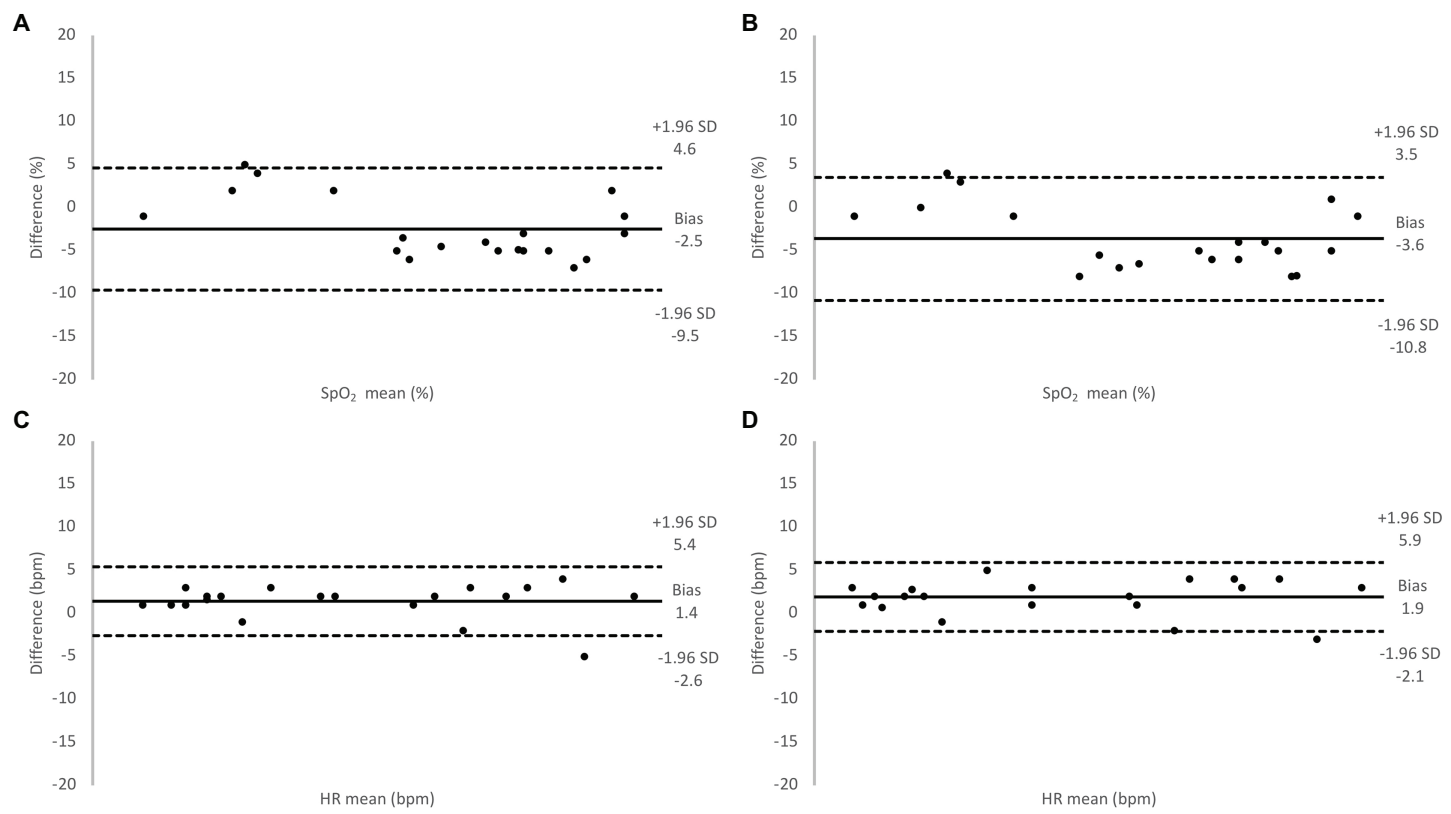
Relative difference between oxygen saturation (SpO_2_) values measured using the Masimo pulse oximeter and the SUB pulse oximeter **(A)**, the Lifesense pulse oximeter and the SUB pulse oximeter **(B)**, and for HR values measured using the Masimo pulse oximeter and the SUB pulse oximeter **(C)**, the Lifesense pulse oximeter and the SUB pulse oximeter **(D)**, under hypoxic conditions.

During baseline measurements on the 20 participants, the two transmission pulse oximeters showed similar values of SpO_2_ and HR as the SUB pulse oximeter (Table 1), and the Bland-Altman analysis showed good agreement between the methods (Figure 5). During the hypoxic episode, the SpO_2_ and HR values of the two transmission pulse oximeters were very similar, while the SUB pulse oximeter slightly overestimated the SpO_2_ and slightly underestimating HR compared to the two transmission pulse oximeters (Table 1). The Bland-Altman analysis showed acceptable agreement between the methods; bias was higher and 95% limits of agreements were wider for both SpO_2_ and HR as compared to baseline (Figure 6).

**Table 1:** Simultaneous measurements of arterial oxygen saturation (SpO_2_) and heart rate (HR) values with two transmission pulse oximeters (Masimo Radical-7 and Lifesense LS1-9R) and the submersible pulse oximeter (SUB); bias, precision, 97.5% outer confidence limits (OCL) and 2.5% inner confidence limits (ICL) are shown for Masimo vs. SUB and Lifesense vs. SUB, respectively.

	Baseline		Hypoxic episode	
	SpO_2_ (%)	HR (bpm)	SpO_2_ (%)	HR (bpm)
**SUB**	**98.4 ± 0.9**	**66.8 ± 12.0**	**89.8 ± 6.8**	**79.6 ± 11.3**
**Masimo**	**97.7 ± 1.8**	**67.8 ± 11.9**	**87.4 ± 4.9**	**81.1 ± 11.1**
Bias	−0.8	1.1	−2.5	1.4
Precision	1.5	1.4	3.6	2.0
OCL	−5.3 – 3.7	−3.1 – 5.3	−13.1 – 8.2	−4.6 – 7.4
ICL	−3.1 – 1.6	−1.1 – 3.2	−7.9 – 3.0	−1.7 – 4.5
**Lifesense**	**97.8 ± 0.9**	**67.7 ± 11.8**	**86.2 ± 5.2**	**81.6 ± 11.3**
Bias	−0.6	1.0	−3.6	1.9
Precision	1.0	1.9	3.7	2.1
OCL	−3.6 – 2.5	−4.7 – 6.6	−14.4 – 7.1	−4.1 – 7.9
ICL	−2.2 – 1.0	−1.9 – 3.9	−9.2 – 1.9	−1.2 – 5.0

Data are presented as mean ± SD.

### Part III: SpO_2_ and HR Patterns in Immersed Static Apnea

**Figure 7:**
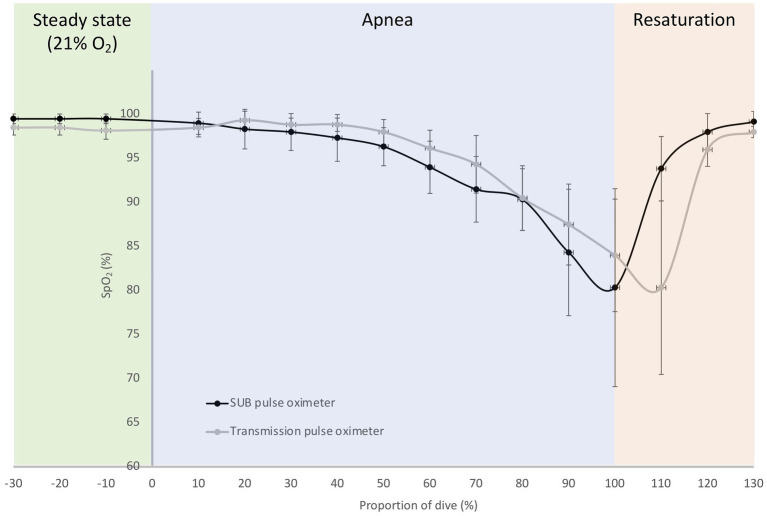
Mean (SD) relative oxygen saturation (SpO_2_) traces during a maximal breath-hold for the SUB pulse oximeter and the Lifesense transmission pulse oximeter; dive time is expressed as a percentage of the total dive duration (184 ± 53 s) for six participants.

**Figure 8:**
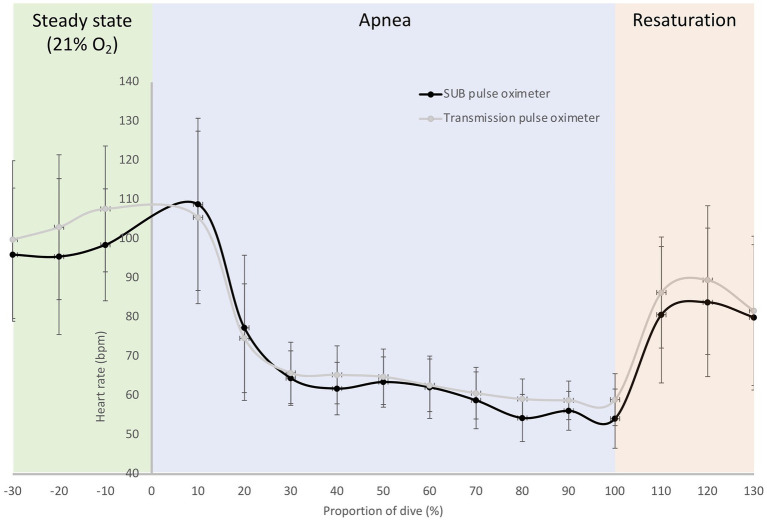
Mean (SD) relative HR traces during a maximal breath-hold for the SUB pulse oximeter and the Lifesense transmission pulse oximeter; dive time is expressed as a percentage of the total dive duration (184 ± 53 s) for six participants.

In the pilot tests on six freedivers, baseline measurements with the SUB pulse oximeter showed an SpO_2_ of 100 ± 0.5% and a HR of 96 ± 17 bpm, while baseline measurements with the Lifesense pulse oximeter showed an SpO_2_ of 98 ± 1% and a HR of 99 ± 20 bpm. The divers performed a maximal static apnea of 184 ± 53 s. During the static apnea, the traces of both pulse oximeters initially showed a relatively similar pattern for SpO_2_, but an earlier drop in SpO_2_ for the SUB pulse oximeter compared to the Lifesense pulse oximeter (Figure 7). The mean SpO_2_ nadir for the Lifesense pulse oximeter (76 ± 10%) occurred later, at 16 ± 8 s after the end of apnea, compared to the SpO_2_ nadir from the SUB pulse oximeter (74 ± 13%), at 1 ± 4 s from the end of apnea (Figure 7). Resaturation was faster for the SUB pulse oximeter (14 ± 7 s) compared to the Lifesense pulse oximeter (26 ± 12 s; Figure 7). The HR traces showed a similar pattern across the apneas for both pulse oximeters (Figure 8). The diving heart rate for the SUB pulse oximeter was 61 ± 5 bpm, and for the Lifesense pulse oximeter it was 62 ± 5 bpm.

## Discussion

Our newly constructed SUB reflective pulse oximeter seemed to function well, as it recorded SpO_2_ and HR in good agreement with the clinical transmission pulse oximeters used, both under dry normoxic and hypoxic circumstances (*n* = 20), and in water (*n* = 6). Our data indicate that the prototype device may respond faster to oxygen desaturation and resaturation during voluntary static apnea compared with the clinical transmission pulse oximeter. The 15 s difference in response time was likely due to the location of the probes rather than to differences in the mechanisms of the technology.

Indeed, reflective pulse oximetry was designed for measurements on alternative locations, such as the forehead (Cheng et al., 1988), but to avoid any possible discrepancies in the displayed results of the different pulse oximeters related to probe location (Clayton et al., 1991; Bell et al., 1999), we chose to attach all probes to fingertips in part II of our study. Under normoxic circumstances, the SUB pulse oximeter consistently overestimated SpO_2_ an average of 0.8% compared to the Masimo pulse oximeter, and 0.6% compared to the Lifesense pulse oximeter, with a relatively similar dispersion (1.5 and 1.0%, respectively), which, in a clinical setting, is likely to be of little importance (Wax et al., 2009). Our results are comparable to a study by Abey and Kyriacou (2015), who used a similar approach to validate a custom-made reflective pulse oximeter. They applied the reflective sensor to the finger and compared its results to the same commercial finger probe as used in our study (Masimo Radical-7), and found that baseline SpO_2_ values were very similar. We thus conclude that when the SUB pulse oximeter is attached to the finger, it produces results in good agreement with other transmission pulse oximeters during baseline measurements.

When our 20 participants switched to hypoxic gas mixture breathing, stable values on all pulse oximeters were obtained after about two and a half minutes (SpO_2_ ~88%) and remained so until the end of the 3-min period. The SUB pulse oximeter consistently overestimated SpO_2_ an average of 2.5% compared to the Masimo pulse oximeter, and 3.6% compared to the Lifesense pulse oximeter, with a similar dispersion (3.6 and 3.7%, respectively), which may be problematic and needs to be solved. Several studies previously investigated the performance of reflective and transmission pulse oximeters under conditions of poor perfusion. Berkenbosch and Tobias (2006) compared arterial blood oxygen saturation ranging from 84.1 to 99.2% from a group of pediatric patients with SpO_2_ from a transmission probe and reflective forehead probe, and found that SpO_2_ from both sensors were in good agreement with actual arterial oxygen saturation (SaO_2_; 1.4 and 0.6% bias, respectively), with similar precision (2.7 and 2.6%, respectively).

This approach was comparable to a study by Hodgson et al. (2009), who reported that the forehead probe was less precise (4.2% dispersion) and deviated more from SaO_2_ than the finger probe (3.4 and 1.1%, respectively), but nevertheless concluded that both pulse oximeters were equally accurate in detecting changes in SaO_2_. Spoorenberg et al. (2016) employed a protocol that resembles the one used in our study, where healthy participants inhaled a hypoxic gas mixture of 15% O_2_, while sitting on a chair, while SpO_2_ was monitored simultaneously with a transmission pulse oximeter at the finger and a reflective pulse oximeter at the forehead. They showed a greater decrease in SpO_2_ measured at the finger compared to the forehead (median SpO_2_ 92 and 95%, respectively).

It must be kept in mind that probe location for the reflective pulse oximeter in these three studies differed from our study, since we applied all probes on the fingers, so the differences in SpO_2_ found in the above-mentioned studies may be attributed to probe location. Nevertheless, since pulse oximeters tend to be less accurate at lower saturations, this small variation between devices seems to be in the same range as previously reported studies (Hodgson et al., 2009; Wilson et al., 2010; Louie et al., 2018). From a diver safety perspective, however, an overestimation of SpO_2_ is perhaps less desirable than a slight underestimation of SpO_2_, as this might increase the margin of error negatively when SpO_2_ decreases even further toward the point when blackout may occur. Accordingly, alterations to the algorithms are necessary to ensure that SpO_2_ is at least not overestimated, and future evaluation studies of the SUB pulse oximeter should include comparisons to the gold standard, i.e., arterial blood oxygen saturation measurements.

For HR in dry conditions in the 20 volunteers, the SUB pulse oximeter underestimated HR during normoxic breathing an average of 1.1 and 1.0 bpm with concomitant precision of 1.4 and 1.9 bpm compared to the Masimo and Lifesense pulse oximeter, respectively. During hypoxic breathing the difference between pulse oximeters remained in the same range, the SUB underestimated HR an average of 1.4 bpm compared to the Masimo, and 1.9 bpm compared to the Lifesense, with similar precision (2.0 and 2.1 bpm, respectively). A discrepancy of less than 10 beats per minute has little clinical value (De Ridder et al., 2018) and, therefore, we consider the quality of the SUB pulse oximeter for measuring HR under dry normoxic and hypoxic circumstances acceptable.

In the static apnea protocol in six freedivers, we did not apply the probes of the SUB pulse oximeter to the fingers, because we wanted to investigate its performance when progressive hypoxemia and peripheral vasoconstriction are both at play. We anticipated a differing oxygen desaturation and resaturation response time by both pulse oximeters when our participants performed a maximal apnea. This is because pulse oximeters of different manufacturers use different algorithms and filtering methods to calculate SpO_2_, so all pulse oximeters exhibit a slightly different response time of oxygen saturation (Trivedi et al., 1997).

We found that the SpO_2_ desaturation and resaturation curves, as well as the HR curves, from both pulse oximeters were similar in duration and minimum displayed SpO_2_ value. However, compared to the SUB pulse oximeter, the SpO_2_ curve of the Lifesense pulse oximeter displayed a temporal “right shift,” thus the SpO_2_ nadir occurred 20 s later. These finding are in line with the results of several previous studies, which also reported a delayed desaturation and resaturation response times for finger pulse oximeters compared to forehead pulse oximetry (Bebout et al., 2001; Choi et al., 2010) and/or arterial blood sampling (Severinghaus et al., 1989; Trivedi et al., 1997).

The differences in the delay of displayed SpO_2_ is most likely not due to the different technology used (reflective or transmissive), but rather occurs as a result of probe location. The blood circulation in the forehead comes from the branch of the supraorbital artery, which arises from the internal carotid artery, so arterial blood reaches the monitoring site of the reflective probe more quickly than the monitoring site of the transmission probe at the finger (Hertzmann and Roth, 1942; Jopling et al., 2002). Ear lobe reflective pulse oximetry, which measures saturation from blood coming from the external carotid artery, has also been suggested as a better alternative for measuring SpO_2_, due to its superior precision (Seifi et al., 2018) and faster desaturation response time (Lindholm et al., 2007) than finger pulse oximeters. However, some studies concluded that forehead reflective pulse oximetry could be the preferred method under conditions of poor perfusion (Fernandez et al., 2007; Shallom et al., 2007) with shorter desaturation response times than ear lobe reflective pulse oximetry (MacLeod et al., 2005).

In addition, transmission pulse oximetry at the finger has been shown to be prone to conditions of low peripheral perfusion (Sugino et al., 2004), and this is likely to occur in static apnea and freediving when the vagally induced “diving response” causes peripheral vasoconstriction (Finley et al., 1979; Fagius and Sundlöf, 1986) and bradycardia (Craig, 1963; Gooden, 1994). This peripheral vasoconstriction is most noticeable at the extremities and comes with a large degree of vasoactivity, which affects pulse oximeter performance (Evens and Geddes, 1988). Indeed, the HR patterns from our freedivers measured with both pulse oximeters showed a clear apnea-induced HR-reduction in response to the start of apnea, and HR remained stable until apnea was terminated, at which point HR increased as the freedivers started to breathe again. It is likely that the delayed desaturation and resaturation response time in apneic activities, as seen in the current study, is enhanced by the combined effects of progressive hypoxemia and peripheral vasoconstriction. When MacLeod et al. (2005) investigated the response times of finger and forehead pulse oximeters, they found that both a short hypoxic challenge and mild hypothermia significantly prolonged the response time of finger pulse oximeters. MacLeod et al. (2005) attributed this delayed response time to the vasoconstrictor response in the finger, which seems to be absent in the blood circulation of the forehead (Hertzmann and Roth, 1942; Jopling et al., 2002). Thus, it seems that the observed difference in response time between the SUB and the Lifesense pulse oximeter can be explained by the difference in probe location, while the underlying mechanism for this difference seems to be related to the travel distance of oxygenated blood from the lungs to the monitoring site and the degree of vasoconstriction at the specific monitoring site. However, as we did not compare measurements of both pulse oximeters applied to the fingers during the static apnea protocol, which would have required an additional protocol to our planned tests, we therefore cannot rule out differences in performance of the pulse oximeters itself as the reason for the delayed response time.

A short pulse oximetry response time is important in critical care, but also in research on apnea and freediving, since oxygen saturation in these situations may drop to dangerously low levels very quickly. Many elite freedivers are able to perform a static apnea until reaching an arterial oxygen saturation of less than 60%, while maintaining consciousness (reviewed by Bailey et al., 2017). As the recreational freedivers used in our study did not desaturate to such a low level, it remains to be investigated to what extent the SUB pulse oximeter is capable of accurately measuring saturations at these levels, while keeping in mind the general limitations of pulse oximetry when SpO_2_ drops below 70% (Chan et al., 2013).

Upon resurfacing after actual freediving in the sea, Fernández et al. (2019) measured nadir SpO_2_ values of around 85% approximately 25 s after resurfacing from 30 m dives using conventional finger pulse oximeters. This study, however, could not provide any information about SpO_2_ during the period of time when the freediver is submerged. Using submersible equipment, in line with our study, Stanek et al. (1993) showed that nine Ama divers only slightly desaturated during short, shallow freedives, and Kuch et al. (2010) demonstrated that a trained freediver desaturated less than an untrained individual during single shallow dives to 10 m. We have now shown that our newly developed prototype pulse oximeter works underwater, in fairly good agreement with commercial devices. By developing the SUB pulse oximeter further, we aim to continue our research by investigating oxygen management and the rate of desaturation under water, during various types of freediving, e.g., during deep freediving in competition divers or when multiple repetitive dives are performed by recreational divers or spear fishers. The new SUB pulse oximeter, found useful to continuously monitor SpO_2_ and HR under water, may enable us to explain the energetic demands in real freediving and possibly predict and subsequently prevent freediving accidents related to hypoxic “blackout.” However, in deep freediving, ambient temperature may decrease substantially, which could disturb the performance of the SUB pulse oximeter due to a possible increase in the degree of vasoconstriction in the freedivers. We therefore aim to conduct future tests in more extreme thermal and hyperbaric conditions to examine the performance and accuracy of the device.

### Study Limitations

A limitation of the current study is the small number of participants in part III, the pilot test on six immersed recreational freedivers, which must be addressed in future larger studies using the SUB pulse oximeter. A future thorough validation study of the SUB pulse oximeter should also include a larger sample size than currently used in part II. Although the raw data from all our trials in part III showed a dropout rate of 18%, which, in clinical settings would not be acceptable, we are satisfied with our results. Many of the missing data points occurred when stable values were obtained before and after the missing data point, and could thus be accounted for with a simple 5 s running median, which consequently resulted in 1% loss of data. However, in some of the participants, loss of data occurred when saturation was low and detection of a proper signal was, apparently, difficult. When the SpO_2_ algorithm worked well, AC changes of the infrared and red signals were only caused by pulsations of the arterial blood. However, in cases where the venous blood volume under the sensor changed, the infrared as well as the red AC and DC signals were affected, while still showing a similar waveform, which accordingly lead to incorrect calculations of SpO_2_. However, our improved algorithm failed as well under these circumstances, as infrared and red signals continued to correlate well. Changes in venous blood volume may have been caused by specific movements, blood pressure changes, involuntary breathing movements or pressure changes of the hood. Accordingly, in some cases, we obtained only a small number of usable values at the end of the dive, and arterial oxygen saturation may have been even lower than those presented here. However, without continuous data, we recognize that further development of the SUB pulse oximeter is necessary to improve data collection for further research in this field. In addition, in both dry and immersed tests, skin temperature should have been monitored, as it could have affected our results. Furthermore, elite freedivers could have been included to challenge the equipment during severe arterial oxygen desaturation. Monitoring SpO_2_ during apnea in general will always need to deal with the effects of peripheral vasoconstriction, as discussed earlier. Although the forehead seems to be less affected by peripheral vasoconstriction than peripheral monitoring sites such as the finger, there will most likely be a delay in both cases, as compared to arterial blood-gas oxygen sampling. Therefore, it should be realized that the method proposed here is most useful for monitoring relevant changes of SpO_2_, rather than to establishing the exact SpO_2_ at a given timepoint, which can only be determined accurately with arterial blood-gas oxygen sampling.

## Conclusion

We concluded that measurements of SpO_2_ and HR can be done with the SUB pulse oximeter on healthy participants during normoxic and hypoxic breathing, as well as during submersed static apnea, and produce similar results as commercially available pulse oximeters. When SpO_2_ is monitored during static apnea, the SUB pulse oximeter, with probe placement on the temples, will respond quicker to changes in oxygen desaturation and resaturation compared to a finger pulse oximeter. The SUB pulse oximeter should be further developed and tested in a range of different conditions with a larger number of freedivers, aiming to examine reproducibility, to limit loss of data, and to improve accuracy in situations when movements, severe hypoxemia and depth may impact signal quality. The further developed monitor could be a great asset in freediving research and could improve freediving safety.

## Data Availability Statement

The datasets presented in this article are not readily available because the human data collected in this study cannot be provided due to conflict with Ethical approval. Requests to access the datasets should be directed to eric.mulder@miun.se.

## Ethics Statement

The studies involving human participants were reviewed and approved by Regional Committee for Medical and Health Research Ethics, Umeå, Sweden. The patients/participants provided their written informed consent to participate in this study.

## Author Contributions

EM contributed to the study design, planning and organization of laboratory, field tests, and procedures, data collection, data analysis, and manuscript writing. ES contributed to the original idea, planning and organization of laboratory, field study tests, and procedures, manuscript writing, and proofreading. AS contributed to the original idea, construction of measurement methods, planning and organization of field study tests and procedures, data collection, manuscript writing, and proofreading. All authors contributed to the article and approved the submitted version.

### Conflict of Interest

The authors declare that the research was conducted in the absence of any commercial or financial relationships that could be construed as a potential conflict of interest.
